# “An Unusual Case of Bilateral Sudden Mixed Hearing Loss with Complete Remission”: A Case Report and Pathophysiological Considerations

**DOI:** 10.3390/reports8030116

**Published:** 2025-07-21

**Authors:** Musat Gabriela Cornelia, Codrut Sarafoleanu, Lucia Radu, Ovidiu Musat, Ionut Tanase

**Affiliations:** Department of Otolaryngology, Faculty of Dentistry, “Carol Davila” University of Medicine and Pharmacy, 030167 Bucharest, Romania; gabriela.musat@umfcd.ro (M.G.C.); codrut.sarafoleanu@umfcd.ro (C.S.); lucia.radu@umfcd.ro (L.R.); ionut.tanase@umfcd.ro (I.T.)

**Keywords:** mixed hearing loss, sudden sensorineural hearing loss, otitis media with effusion, viral labyrinthitis

## Abstract

**Background:** Sudden-onset bilateral mixed hearing loss in adults is an extremely rare condition but challenging to diagnose and treat. Conductive hearing loss is associated with otitis media, while the simultaneous presence of a sensorineural component requires supplementary investigation for possible shared pathophysiological mechanisms. **Case Presentation:** We report the case of a 41-year-old male who was admitted to our hospital with a 48 h history of bilateral, fast progressive hearing loss following a viral illness. The audiologic testing revealed bilateral severe mixed hearing loss. Tympanometry indicated the presence of middle-ear effusion, and myringotomy confirmed the existence of pressurized serous fluid. Treatment consisted of systemic and intratympanic corticosteroids, antibiotics, and supportive therapy. The patient had an unexpected full recovery of auditory function within one month. **Discussion:** Multiple hypotheses were considered. We hypothesized the coexistence of unrelated conductive and sensorineural hearing loss or a unifying pathological process. Theories discussed include a direct viral insult to the cochlear structures or even pressure-mediated damage to the basal cochlea due to the simultaneous inward displacement of the oval and round windows. The complete resolution of hearing loss is the indicator of a reversible etiology, possibly due to transient inner ear dysfunction secondary to middle-ear pathology or viral infection. **Conclusions:** This case illustrates the complexity of diagnosing acute mixed hearing loss. This report emphasizes a rare case of sudden-onset bilateral mixed hearing loss with a complete recovery, contributing valuable insight into under-reported and diagnostically complex presentations.

## 1. Introduction

Hearing loss is one of the most common sensory impairments, affecting approximately 18% of the population [1]. Sudden hearing loss is typically characterized by a rapid onset of sensorineural hearing impairment occurring in less than 72 h. Unilateral sensorineural hearing loss is relatively well-documented and often idiopathic. Bilateral involvement is far less common and may be an indicator of an underlying systemic or otologic pathology. Mixed hearing loss, featuring both sensorineural and conductive components, is extremely rare in acute presentations. Bilateral sudden mixed hearing loss is exceedingly uncommon. In this report, we present a unique case of sudden-onset bilateral mixed hearing loss in a healthy individual, with no preceding trauma or ototoxic exposure. To our knowledge, only one other similar case has been reported in the literature, underscoring the rarity and clinical intrigue of this presentation [2].

### Clinical Significance

The recognition and prompt evaluation of bilateral sudden mixed hearing loss is important because there is a potential for underlying systemic conditions and the risk of permanent auditory impairment. This case underlines the importance of considering both sensorineural and conductive components when diagnosing and managing sudden hearing loss, especially when bilateral symptoms are present. This report aims to increase the limited knowledge of this rare presentation. Detailed clinical work-up and audiometric testing are important in identifying atypical patterns.

## 2. Case Presentation

We report the case of a 41-year-old male who presented to our hospital with bilateral, rapidly progressive hearing loss that had developed over a period of 48 h. Three days prior to the onset of auditory symptoms, the patient reported a mild viral illness. He declared that his hearing was normal before this episode. There was no personal or family history of otologic disease.

On clinical examination, the patient appeared extremely anxious and distressed. He reported sudden bilateral, important hearing loss with only mild otalgia, localized to the right ear. Communication with the patient required a significantly raised vocal volume due to the severity of his hearing impairment. Vital signs, including heart rate and blood pressure, were within normal limits. Chest radiography was unremarkable, and complete blood count (CBC) did not reveal leukocytosis. However, inflammatory markers, including C-reactive protein (CRP = 10.91), fibrinogen (648mg/dL), and erythrocyte sedimentation rate (ESR = 43), were elevated.

Otoscopic examination revealed bilateral retracted tympanic membranes, with mild hyperemia of the malleus handle on the right side. The tympanic membranes were opacified and lacked the typical light reflex. The retraction of the tympanic membranes was attributed to negative middle-ear pressure caused by Eustachian tube dysfunction during the viral illness. The middle-ear fluid accumulation likely resulted from inflammation-induced drainage impairment.

**Figure 1:**
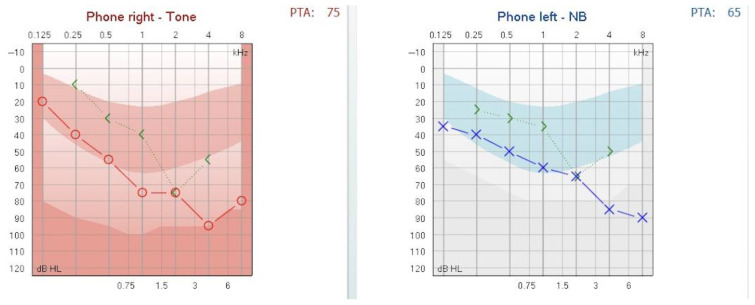
Pure-tone audiogram at presentation showing mixed hearing loss. Moderate hearing loss, 2nd degree, in the left ear and severe hearing loss, 1st degree, in the right ear.

Audiological assessment was performed using an Interacoustics clinical audiometer. Pure-tone audiometry demonstrated mixed hearing loss with a pure tone average (PTA) of 65 dB HL in the left ear and 75 dB HL in the right ear (see Figure 1). Air conduction thresholds were evaluated from 125 to 8000 Hz, and bone conduction thresholds from 250 to 4000 Hz. The air-bone gap analysis for both ears is synthesized in Table 1.

**Figure 2:**
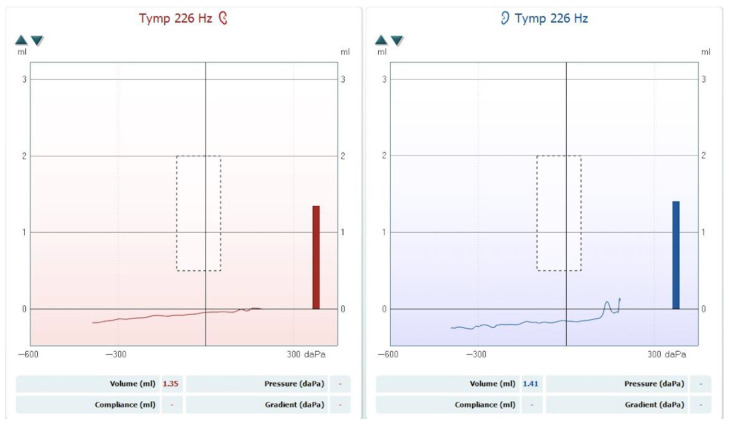
Tympanogram showing bilateral Type B curves consistent with middle-ear effusion.

The hearing loss was classified according to the BIAP recommendations [3]. The patient was diagnosed with moderate hearing loss, 2nd degree, in the left ear and severe hearing loss, 1st degree, in the right ear. A tympanometric evaluation was performed using a Titan tympanometer (Interacoustics), employing a 226 Hz probe tone. The tympanogram showed flat (Type B) curves in both ears, consistent with middle-ear effusion (see Figure 2). Tympanometric results were interpreted based on the static admittance criteria and classified according to the system proposed by Jerger and Liden [4,5].

Upon evaluation, no vestibular signs or symptoms were reported or observed. The patient denied experiencing vertigo, imbalance, or unsteadiness. Clinical examination revealed no nystagmus, positive Romberg sign, or gait abnormalities, suggesting no involvement of the vestibular system.

The patient was initiated on intravenous corticosteroids, antibiotics, vitamins, vasodilators, and nasal decongestants. A myringotomy was subsequently performed, followed by the drainage of a large amount of pressurized serous fluid. The myringotomy was performed on day 1 of admission, shortly after audiologic and tympanometric evaluation.

We injected steroids into each ear. The patient received intratympanic dexamethasone 0.5 mL per ear at a concentration of 4 mg/mL, equating to 2 mg per ear. This dose was administered once, immediately following the myringotomy. Additionally, the patient received systemic methylprednisolone 500 mg/day intravenously for 5 days.

**Figure 3:**
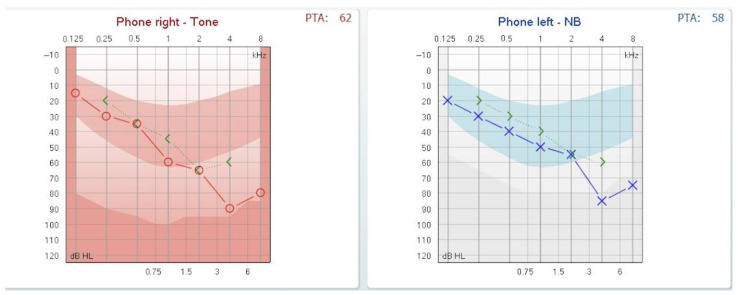
Audiogram on day 2 post-myringotomy showing closure of the air-bone gap.

The second day after the myringotomy, the audiogram showed a marked decrement of the air–bone gap as presented in Figure 3.

**Figure 4:**
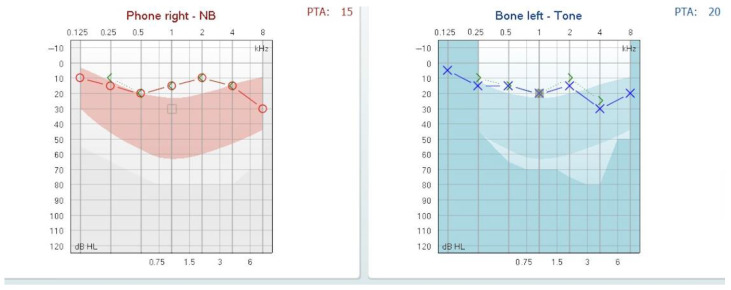
Follow-up audiogram at one month showing normalized auditory thresholds.

One month post-treatment, the patient achieved recovery of auditory function, with the normalization of hearing thresholds as demonstrated in follow-up audiometry (see Figure 4).

Given the patient’s rapid recovery and resource constraints, a comprehensive systemic workup—including contrast-enhanced MRI and serological testing (e.g., autoimmune panel, syphilis, HIV)—was not performed. We recognize this as a limitation and acknowledge the importance of such diagnostic evaluations in similar cases.

## 3. Discussion

The case we presented is particularly interesting due to its rarity. It offers an opportunity to explore possible pathophysiologic mechanisms. The bilateral occurrence of sudden mixed hearing loss is exceptionally rare, with only one similar case documented in the literature.

Initially, we thought that the patient may have had pre-existing hearing loss that had gone unnoticed, and that we were witnessing an aggravation of a pre-existing hypoacusis. The second audiogram we performed strengthened our hypothesis that the patient had a previous sensorineural hearing loss aggravated by a concurrent acute serous otitis. The fact that the patient experienced total recovery after treatment made us doubt this initial thinking.

We started by admitting two major lines of reasoning. One stated that there is no connection between the two types of hearing loss, and the mixed type is just a coincidence. An alternative line of thinking is that there is a connection between the two types of hearing loss, potentially explained by a pathological process affecting both the middle and inner ear.

First hypothesis. Theoretically, the conductive hearing loss may have preexisted, with a newly developed sensorineural component superimposed on it. However, epidemiological data indicate that conductive hearing loss with an intact tympanic membrane in adult males is relatively uncommon. Potential causes of chronic conductive hearing loss with an intact tympanic membrane include otosclerosis [6], ossicular chain fixation/dislocation [7], and chronic serous otitis media. Otosclerosis and ossicular chain fixation are typically associated with a type A tympanogram, making these diagnoses less likely in this case. Chronic serous otitis media remains a possible explanation, as it is characterized by a type B tympanogram [8], but the history of the disease is not consistent with this hypothesis.

Sudden sensorineural hearing loss (SSNHL) is defined as a rapid-onset hearing loss of at least 30 decibels (dB) across 3 contiguous frequencies occurring within a 72 h period [9,10]. It typically affects one ear (unilateral) and presents without warning, often accompanied by tinnitus, aural fullness, or vertigo. SSNHL is considered an otologic emergency due to the potential for permanent hearing damage. The annual incidence of unilateral SSNHL is estimated to be between 5 and 20 cases per 100,000 individuals, though the true prevalence may be under-reported due to spontaneous recovery in some cases [11,12]. The etiology remains idiopathic in approximately 85% of cases, but potential causes include viral infections, vascular compromise, autoimmune disorders, and cochlear membrane ruptures.

Bilateral SSNHL is significantly rarer. Epidemiologically, the exact incidence of bilateral SSNHL is not well-established due to its rarity. Different studies report varying incidences of the disease from 0.57 to 14.5% of all SSNHL cases [13,14,15]. It is typically more concerning due to its association with systemic pathology. Unlike unilateral SSNHL, bilateral cases are more frequently linked to identifiable underlying conditions and often warrant comprehensive systemic evaluation. Causes of bilateral SSNHL include autoimmune inner ear disease, infections (such as syphilis or HIV), neoplastic processes (like bilateral vestibular schwannomas in neurofibromatosis type II), vascular insults, and exposure to ototoxic agents [16,17]. Still, its sudden presentation and potential for profound auditory impairment make prompt diagnosis and management critical.

As a conclusion for this hypothesis, we considered the possibility that a sudden-onset bilateral sensorineural hearing loss occurring in conjunction with a chronic conductive component is extremely low and could be ruled out.

Second hypothesis. Another hypothesis we took into account was that the patient had a pre-existing sensorineural hearing loss, which was then compounded by serous otitis. The prevalence of sensorineural hearing loss is significant. According to the WHO in 2021, globally, over 5% of the world’s population (around 430 million people) have disabling hearing loss, with the majority being sensorineural. The prevalence of serous otitis media in adults is notably lower than in children but is still clinically significant. The incidence of adult otitis media with effusion varies between 3.2 and 6.2% in a study from 2023. The male sex and unilateral ear involvement are more common [18]. Considering this, the possibility of a coincidence was low but still possible, so the hypothesis that the patient had an unrecognized sensorineural hearing loss associated with an acute serous otitis was considered plausible. The viral episode that preceded the sudden onset of the hearing loss supports the possibility of a diagnosis of acute otitis media with effusion.

However, the patient reported normal hearing before the viral episode, experienced a sudden onset of symptoms, and subsequently had a complete recovery from the hearing loss. These factors ruled out our hypothesis that the patient had a previously unrecognized sensorineural hearing loss. Consequently, we were left searching for an explanation for the acute onset of a mixed hearing loss.

Third hypothesis. The next hypothesis was that there was only one pathophysiological process affecting both the middle and the inner ear simultaneously.

The diagnosis of otitis media with effusion was confirmed based on the appearance of the tympanic membrane, the audiogram showing a conductive component, the bilateral type B tympanogram, and the characteristics of the fluid drained from the middle ear during myringotomy. Is it possible that the sudden sensorineural hearing loss is concomitant with the serous otitis in both ears, totally unrelated?

Another question that arose concerned the pathophysiological process responsible for both the conductive and sensorineural components of the hearing loss. While the conductive component was readily explained by the presence of serous otitis, which typically causes this type of hearing loss, the sensorineural component was more difficult to account for. How can this be explained?

Conductive hearing loss in otitis media appears when the mechanical transmission of sound through the middle ear is impaired because fluid in the middle ear does not allow the efficient mechanical transmission of sound vibrations through the tympanic membrane and ossicles to the inner ear. The inner ear and neural pathways are typically intact.

The oval window and round window are essential structures in the ear that facilitate the transformation of sounds into fluid motion inside the cochlea. The round window membrane moves in the opposite phase to the oval window. This opposing motion helps accommodate the fluid displacement within the cochlea, enabling unobstructed perilymph movement and optimizing sound transmission [19].

As the vibrations pass through the cochlear fluid, they cause displacement of hair cells within the organ of Corti. This organ is tonotopically organized: the area near the base of the cochlea, close to the oval window, detects high-frequency sounds, while the area at the apex, near the helicotrema, responds to low-frequency sounds.

Blocking of the oval window will result in conductive hearing loss, as it typically happens in otosclerosis. Blocking of the round window is also followed by conductive hearing loss, as it does not allow the fluid inside the inner ear to move outward.

The increase in endolymph volume within the inner ear, characteristic of Ménière’s disease, leads to sensorineural hearing loss. The increment of the endolymph volume generates an increment of the pressure. This pressure particularly affects the apex of the cochlea, where pressure changes are most pronounced because of the smaller spaces. As a result, the audiogram typically shows sensorineural hearing loss in the lower frequencies, which are processed near the cochlear apex.

A question that might arise is whether, in cases where the volume of the endolymph remains normal, the sensorineural hearing loss affecting high frequencies might be explained by pressure exerted near the base of the cochlea. We hypothesize that increased middle-ear pressure could simultaneously force both the oval and round windows inward. This abnormal inward displacement may transmit excessive mechanical stress to the basal turn of the cochlea, where high-frequency sounds are processed, potentially resulting in a sloping sensorineural hearing-loss pattern on the audiogram. This is just a supposition we are making, not supported by the fact that releasing the pressure in the middle ear by myringotomy did not automatically solve the sensorineural component, as we can see in Figure 3.

The hypothesis that was finally accepted was that of a viral infection that may have affected the cells of the Corti organ simultaneously, thereby explaining the sensorineural component of the hearing loss. Viral labyrinthitis is a possible explanation, but we should also emphasize the fact that the patient had no vestibular symptoms or signs.

Indirect cochlear insult due to inflammatory mediators or toxic byproducts is released during the immune response to a viral infection. This mechanism may account for the reversible sensorineural component through inflammatory pathways rather than direct viral cytotoxicity.

In a study from 1972, Paparella et al. studied the impact of otitis media on cochlear function. They proposed the hypothesis that the infection spreads from the middle ear to the inner ear via the round window [20]. The study was focused mainly on chronic otitis media, but the changes might happen even in acute otitis. The modifications of the round window membrane and the basal tour of the cochlea were considered significant for the impact on the sensorineural component of the hypoacusis. This phenomenon was mainly present in suppurative otitis media. The link between chronic otitis media and sensorineural hearing loss is already recognized [21,22]. A study from 2014 on the association of acute otitis media in adults with sensorineural hearing loss found that in approximately 9,3% of the ears with acute otitis media, there was also sensorineural hearing loss [23]. Serous otitis media is not usually associated with sensorineural hearing loss. Mutlu et al. studied the association between otitis media with effusion and sensorineural hearing loss in children [24]. They considered any bone conduction loss higher than 25 dB at any of the frequencies from 250 to 4000 Hz as sensorineural hearing loss. They found that 9% of the ears with otitis media with effusion had associated sensorineural hearing loss, but in the cases they presented, the bone conduction decrement was not as important as in our patient.

A systematic review from 2021 studying the correlation between acute recurrent otitis media or chronic suppurative otitis media and sensorineural hearing loss established the fact that no conclusion on this correlation can be made [25]. The concurrence of a serous otitis with sensorineural hearing loss is, nevertheless, very rare. Bilateral serous acute otitis media associated with sensorineural hearing loss is even rarer. We already mentioned the fact that we found only one documented case of mixed sudden hearing loss in the literature.

This clinical picture is consistent with serous labyrinthitis, likely arising as a complication of a viral middle-ear infection. Notably, the lack of vestibular symptoms is an interesting feature in this case.

## 4. Conclusions

A comprehensive search of the literature revealed only one comparable case—described by Piłka et al. [1]—featuring bilateral mixed hearing loss in a post-viral context. While both cases share features such as acute onset, bilateral involvement, and a viral prodrome, our case is distinguished by the rapid and complete resolution of both conductive and sensorineural components without immunosuppressive treatment. This suggests a transient, likely inflammatory, pathophysiological mechanism, potentially involving both the middle and inner ear structures, consistent with acute serous otitis complicated with serous labyrinthitis.

The absence of additional similar cases in the literature highlights the rarity of this presentation. We believe that this report contributes valuable insight to the diagnostic approach in cases of sudden mixed hearing loss, particularly in distinguishing among autoimmune, infectious, and inflammatory etiologies. Our findings may encourage further reporting and investigation of such presentations, aiding in the refinement of diagnostic and therapeutic strategies.

We propose three plausible mechanisms to explain the sensorineural component: (1) pressure-mediated transmission via the round and oval windows; (2) direct viral cochlear involvement; and (3) inflammatory mediator-related cochlear damage. The overall picture is compatible with serous labyrinthitis, a reversible inflammatory condition involving inner ear structures.

The complete recovery of hearing after intervention with corticosteroids and myringotomy favors the theory of a reversible, inflammation-related etiology.

This case underscores the importance of considering multifactorial mechanisms in atypical presentations of hearing loss and illustrates how a combination of clinical, audiological, and therapeutic data can guide diagnosis and management. Prompt recognition and treatment are essential to achieving favorable outcomes in such complex cases.

## Figures and Tables

**Table 1:** Audiogram air-bone gap analysis for both ears.

Frequency (Hz)	Right Ear AC (dB HL)	Right Ear BC (dB HL)	Right Ear ABG (dB)	Left Ear AC (dB HL)	Left Ear BC (dB HL)	Left Ear ABG (dB)
250	30	15.0	15.0	40	25.0	15.0
500	40	25.0	15.0	50	35.0	15.0
1000	55	35.0	20.0	55	40.0	15.0
2000	70	50.0	20.0	55	35.0	20.0
3000	75			70		
4000	80			80		
6000	70			85		

## Data Availability

The original contributions presented in this study are included in the article. Further inquiries can be directed to the corresponding author.
